# Li and Na Adsorption on Graphene and Graphene Oxide Examined by Density Functional Theory, Quantum Theory of Atoms in Molecules, and Electron Localization Function

**DOI:** 10.3390/molecules24040754

**Published:** 2019-02-19

**Authors:** Nicholas Dimakis, Isaiah Salas, Luis Gonzalez, Om Vadodaria, Korinna Ruiz, Muhammad I. Bhatti

**Affiliations:** 1 Department of Physics and Astronomy, University of Texas Rio Grande Valley, Edinburg, TX 78539, USA; 2 Achieve Early College High School, McAllen, TX 78501, USA; isaiahx10@outlook.com; 3 PSJA Thomas Jefferson T-STEM Early College HS, Pharr, TX 78577, USA; luisgpec1@gmail.com; 4 The Science Academy, Mercedes, TX 78570, USA; omnirajvadodaria@gmail.com; 5 South Texas High School for Health Professions, Mercedes, TX 78570, USA; korinna_ruiz@yahoo.com; 6 Department of Physics and Astronomy, University of Texas Rio Grande Valley, Edinburg, TX 78539, USA; muhammad.bhatti@utrgv.edu

**Keywords:** graphene, quantum theory of atoms in molecules, electron localization function, density functional theory, Li, Na

## Abstract

Adsorption of Li and Na on pristine and defective graphene and graphene oxide (GO) is studied using density functional theory (DFT) structural and electronic calculations, quantum theory of atoms in molecules (QTAIM), and electron localization function (ELF) analyses. DFT calculations show that Li and Na adsorptions on pristine graphene are not stable at all metal coverages examined here. However, the presence of defects on graphene support stabilizes both Li and Na adsorptions. Increased Li and Na coverages cause metal nucleation and weaken adsorption. Defective graphene is associated with the presence of band gaps and, thus, Li and Na adsorptions can be used to tune these gaps. Electronic calculations show that Li– and Na–graphene interactions are Coulombic: as Li and Na coverages increase, the metal valences partially hybridize with the graphene bands and weaken metal–graphene support interactions. However, for Li adsorption on single vacancy graphene, QTAIM, ELF, and overlap populations calculations show that the Li-C bond has some covalent character. The Li and Na adsorptions on GO are significantly stronger than on graphene and strengthen upon increased coverages. This is due to Li and Na forming bonds with both carbon and oxygen GO atoms. QTAIM and ELF are used to analyze the metal–C and metal–metal bonds (when metal nucleation is present). The Li and Na clusters may contain both covalent and metallic intra metal–metal bonds: This effect is related to the adsorption support selection. ELF bifurcation diagrams show individual metal–C and metal–metal interactions, as Li and Na are adsorbed on graphene and GO, at the metal coverages examined here.

## 1. Introduction

Rechargeable lithium ion batteries (LIB) serve as energy storage devices for a variety of applications, such as portable electronics and hybrid and electric vehicles [1,2,3,4]. The LIB performance depends on the structure and composition of the anode and cathode electrodes [5]. Graphite has been used as anode material for LIB [6], with graphite’s theoretical capacity limit set at 372 mAh/g (intercalation compounds of LiC_6_ configurations) [7]. Current and future needs dictate the search for LIB materials with capacities significantly higher than graphite to satisfy portable electronic devices’ requirements [5]. The use of other carbon materials, such as graphene [8,9], C_60_, and carbon nanotubes, increases the LIB capacity to 540 mAh/g, 730 mAh/g, and 784 mAh/g, respectively [10]. Graphene is a monolayer honeycomb lattice of carbon atoms and is known for its high mechanical strength and high thermal and electrical conductivity [11,12,13]. Graphene B and N-doping further increase the LIB capacity to 1227 mAh/g and 872mAh/g, respectively [14].

Increased LIB graphene capacities relative to graphite seem indicative of increased Li atoms being adsorbed on graphene relative to graphite. However, the contrary has been observed via in situ Raman spectroscopy [15]. Although computational simulations support the adsorption of single Li atoms on graphene (one side [16,17,18] and both side adsorption [5,19]), this adsorption is less stable relative to bulk metallic Li. Lee et al. showed that Li adsorption on pristine graphene is not favorable relative to bulk metallic Li under any configuration and is only possible on graphene defects and edges [20]. Okamoto further supported Li adsorption on defects relative to edges [21]. This is due to the edges of the anode LIB materials being covered by low electron conductivity solid electrolyte interphase, thus affecting the reversibility of Li movement during charge and discharge processes. Li adsorption on defective graphene has been reported using computational calculations [21,22,23,24]. The presence of double carbon vacancies on N-doped graphene support can also improve LIB performance [25].

The main disadvantage of LIBs is Li cost, which has been rising steadily in recent years, with the exception of a price drop observed in the last quarter of 2018 [26]. Li is a very rare element (Li concentration is ~35 ppm in the upper continental crust [27]), and thus there are concerns that Li may not be sufficient for meeting future energy demands. LIB, as well as sodium ion batteries (NIB) [26,28], suffer from dendrite growth due to metal nucleation (i.e., clustering), which lowers their performance [1,29]. Li forms clusters due to the small Li diffusion barrier on graphene support [30]. Li cluster formation on pristine graphene (i.e., graphene without defects) and defective graphene [21] has been explored using density functional theory (DFT) [30,31]. Defective graphene is produced during graphene oxide’s (GO) thermal reduction process, as hydroxyl and epoxide O atoms are removed from the GO sheet, via oxidation and reduction [32,33]. Li_6_ clusters collapse during adsorption on 24 vacancies defective graphene [21].

Sodium ion batteries could serve as alternative batteries due to the abundance of Na. However, the NIB capacity of graphite is only 35 mAh/g, substantially lower relative to LIB [26]. This is attributed to NaC_64_ formation [34,35] due to the large Na^+^ size relative to Li^+^ (100 pm vs. 76 pm) and Na high ionization potential [21]. NIB capacities were recorded at 1450 mAh/g, for Na adsorption on defective graphene with divacancy [24]. DFT calculations on Na adsorbed on defective graphene have been reported [36]. Specifically, Na cluster formation for adsorption on pristine and defective graphene has been explored via DFT calculations by Liang et al. [37].

In this work, we examine Li and Na adsorptions on pristine and defective graphene, as well as on graphene oxide (GO), at various metal coverages, using periodic DFT, with and without long-range interactions. The metal-support interactions are elucidated the via changes in the adsorption and cohesive energies (*E_ads_* and *E_c_*, respectively) per metal, the metal–Carbon plane distances (height, *h*), and the electronic charge transfers from the metals to graphene and GO supports. The *E_ads_* and the $E_{c}$ for the adsorption of n-metals (*n* = 1, 2, …) on graphene and GO are defined as

(1) $$E_{ads}=(E_{n\cdot X/support}-n\cdot E_{X}-E_{support})/n$$

(2) $$E_{c}=(E_{n\cdot X/support}-E_{n\cdot X}-E_{support})/n$$

where X is the metal adsorbate and the support is either graphene or GO. The *E_ads_* is the minimum energy required to remove a single Li or Na atom during adsorption on graphene and GO, whereas *E_c_* measures the cluster-support interaction. For *n* = 1, *E_ads_* = *E_c_*. Single metals and their clusters form a bond with the support if *E_c_* < 0. The metal-support interaction is considered stable if the |*E_ads_*| is larger than the metal binding energy of its bulk. The metal-support interactions are also examined via electronic structure analyses.

The local metal–C and the metal–metal interactions are examined by two independent analyses: The quantum theory of atoms in molecules (QTAIM) and the electron localization function (ELF). QTAIM was developed by Bader and co-workers, who note that orbitals, much less wave functions, are unphysical in nature; thus, chemical bonds are not quantum observables [38,39]. However, the electron densities ($\rho \left(\vec{r}\right)$) and their gradients are quantum observables and can be used to characterize chemical systems [39,40]. ELF [41] is a measure of the probability of finding an electron in the vicinity of another same-spin electron. It reveals core, binding, and lone electron pairs in molecular and crystalline systems. Results from both QTAIM and ELF analysis are basis-set and method-independent, as long as a minimally adequate basis set is used in these calculations [42,43].

## 2. Computational Methods

### 2.1. Periodic Structure Modeling 

**Figure 1:**
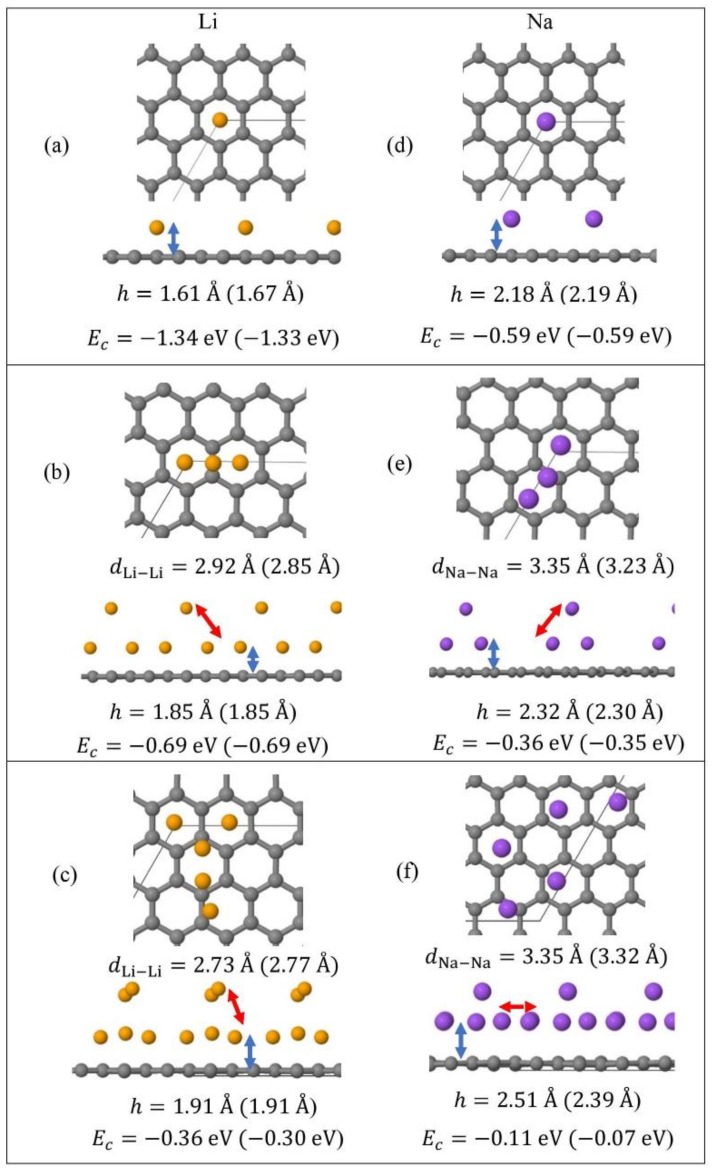
Optimized geometries, heights *h* (blue arrows) for the metal (Li, Na) closest to graphene plane, the closest metal–metal distances (red arrows), and *E_c_* for Li_n_ (yellow spheres) (**a**–**c**) and Na_n_ (purple spheres) (**d**–**f**) (*n* = 1, 3, 5) adsorbed on pristine graphene (C, gray spheres). Values in parentheses include D2 correction. The thin black line is the unit cell boundary.

**Figure 2:**
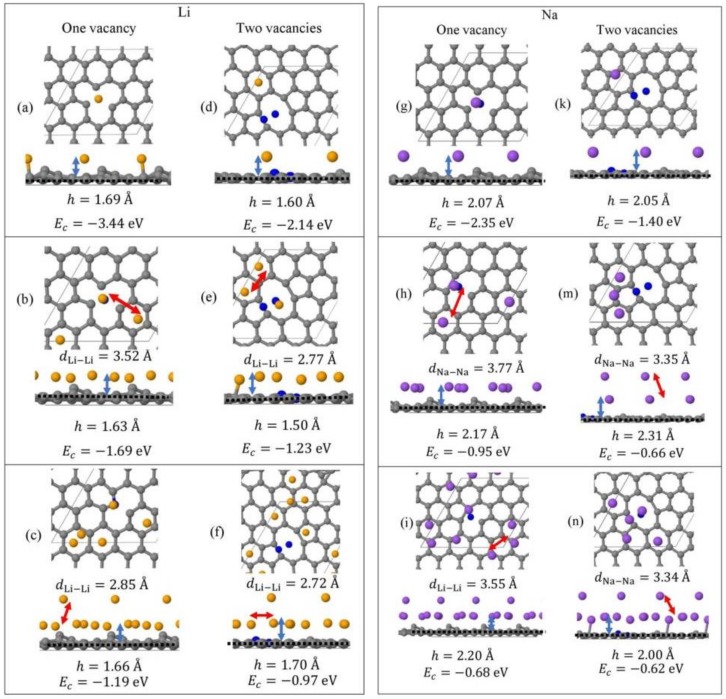
Optimized geometries, heights *h* (blue arrows) for the metal (Li, Na) closest to graphene plane, the closest metal–metal distances (red arrows), and *E_c_* for Li_n_ (yellow spheres) (**a**–**f**) and Na_n_ (purple spheres) (**g**–**n**) (*n* = 1, 3, 5) adsorbed on defective graphene (C, gray spheres, “ghost” C, blue spheres) with one and two C vacancies. All calculations include D2 correction. The thin black line is the unit cell boundary. The thick dashed black line is the carbon plane.

**Figure 3:**
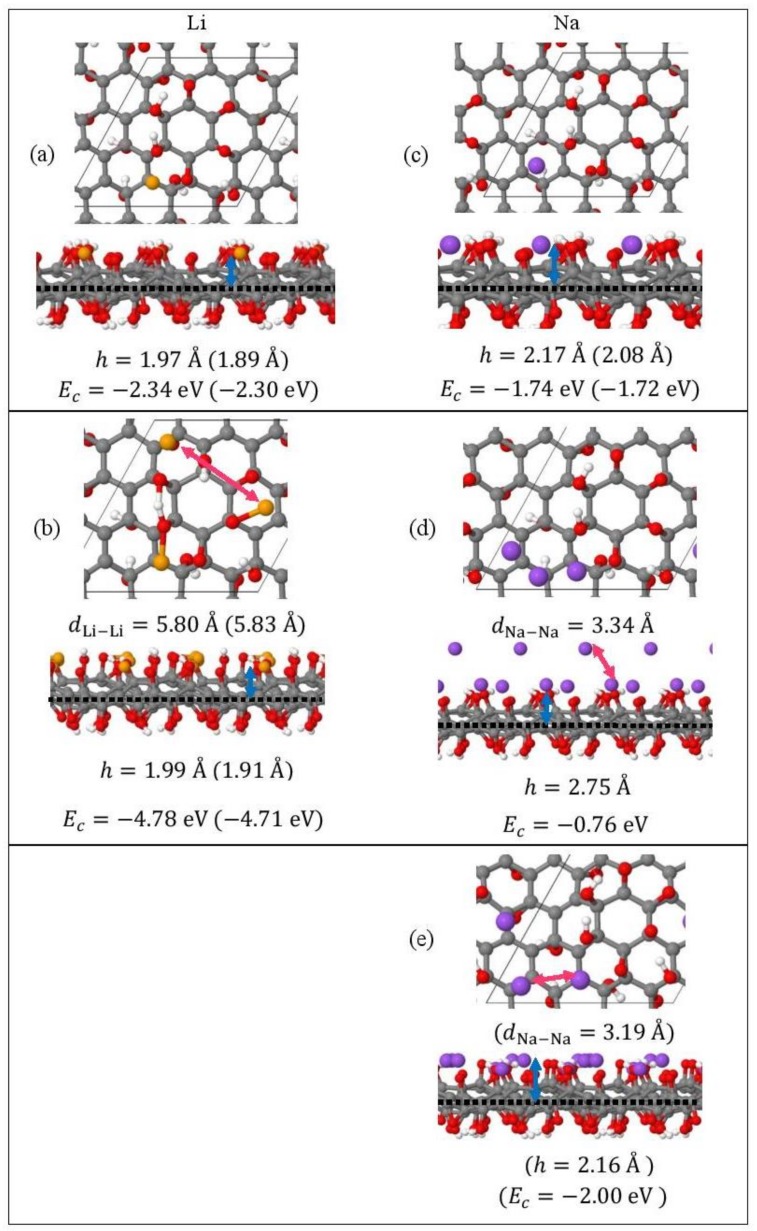
Optimized geometries, heights *h* (blue arrows) for the metal (Li, Na) closest to the C plane, the closest metal–metal distances (red arrows), and *E_c_* for (**a**) Li_n_ (yellow spheres) and (**b**) Na_n_ (purple spheres) (**c**–**e**) (*n* = 1, 3) adsorbed on GO (C, gray spheres; O, red spheres; H white spheres). Values in parentheses include D2 correction. The thin black line is the unit cell boundary. The thick dashed black line is the carbon plane.

Monolayer pristine graphene is modeled as a two-dimensional 4 × 4 hexagonal lattice with 32 carbon atoms in the unit cell. Figure 1 shows the DFT optimized structures for Li_n_ and Na_n_ (*n* = 1, 3, 5) adsorbed on pristine graphene, the Li and Na *E_c_* values, the heights *h* for the closest metal to the graphene plane, and the smaller of the metal–metal distances per configuration. The Li_n_/graphene and Na_n_/graphene (*n* =1, 3, 5) unit cells are built as follows: for *n* = 1, single metal atoms (i.e., Li and Na) are initially placed distant from graphene, followed by geometry optimizations; for *n* = 3, two metal atoms are placed distant from the previously optimized Li/graphene and Na/graphene structures, followed by geometry optimizations. A similar approach is followed for the Li_5_/graphene and Na_5_/graphene cases. Figure 2 shows the optimized structures for Li_n_ and Na_n_ (*n* =1, 3, 5) adsorbed on single and double vacancy defective graphene, as well as the *E_c_* and geometrical parameters, in a similar fashion as in Figure 1. Graphene defects are modeled by the use of “ghost” massless C atoms in the graphene lattice: These atoms share the same basis set as C, in agreement with our past report [44]. Here, the ghost atoms are placed in the center of the supercell and the defect is periodically repeated by the use of translational symmetry vectors [45]. Ghost atoms remain fixed in the locations occupied by the original C atoms. Figure 3 shows the optimized structures for Li_n_ and Na_n_ (*n* = 1, 3) adsorbed on graphene oxide (GO, O/C = 43.75%), with *E_c_* and geometrical parameters. Here, GO supports contain epoxides and hydroxyls at 25% and 18.75%, respectively (percentages relative to C). Our GO structure is in agreement with the proposed non-hydrated GO structure from Zhou and Bongiorno [46] (26% and 18% for epoxides and hydroxyls, respectively), found by using X-ray photoelectron spectroscopy and DFT.

### 2.2. DFT Parameters

CRYSTAL17 [47] is used to calculate the optimized geometries and electronic properties for Li and Na atoms adsorbed on graphene (with and without vacancies) and GO. An advantage of CRYSTAL is its treatment on two-dimensional slabs, without the need for artificial periodicity perpendicular to the surface, as is typically found in other DFT codes [48]. Periodic restricted DFT is used here, as in our past work [17,44,49,50,51]. The PBE0 non-empirical/parameter-free functional is used [52,53]. All calculations are treated using the D2 semiempirical correction by Grimme [54]. This correction improves the DFT functionals description of the long-range electron correlations, which are responsible for van der Waals interactions between the adsorbed metals and graphene/GO supports. Moreover, for selective cases, our calculations are repeated without employing D2 corrections. All atoms are described by all-electron basis sets. The C and H atoms are treated by the 3-ζ valence plus polarization basis set (pob-TZVP) from Peintinger et al. [55] and the O atoms by the split-valence 8-411G(2d1f) basis set from Mahmoud et al. [56]. These basis sets are optimized for crystalline calculations. The Li and Na are described by the atomic basis sets (6s3p1d) and (9s5p1d), respectively, and were used in our past work [17]. Calculations are at 0 K, as expected. Temperature and pressure effects will be described in a future work.

For geometry optimizations, Brillouin zone integrations (Monkhorst-Pack grid) [57], the Fermi energy, and the density matrix calculations (Gilat grid) [58,59] are performed on a 12 × 12 grid. The densities of states (DOS) and the electronic band structures are obtained using the denser 48 × 48 grid. The band structures are calculated on the reciprocal space Brillouin zone path Γ-Μ-Κ-M´-Γ, where Γ = (0, 0, 0), Μ = (1/2, 0, 0), K= (2/3, 1/3, 0), and M´ = (0, 1/2, 0). The Fermi surface is smeared with a Gaussian of 0.005 Hartrees for convergence purposes. Moreover, the SCF energy convergence is achieved using Anderson quadratic mixing [60], coupled with additional mixing of the occupied with the virtual orbitals. The SCF energy threshold value for our calculations is set at 10^-9^ Hartrees (default value is 10^-7^ Hartrees). A large integration grid is used: this is a pruned grid with 75 radial and 974 angular points. The Crystal Orbital Overlap Populations (COOP) [61], for selected pairs of atoms, are calculated directly by CRYSTAL17. Charge transfers are calculated using Mulliken population analysis [62] and the iterative Hirshfeld population [63], the latter being based on the atoms in molecules (AIM) approach [38,39]. Hirshfeld populations are less basis-set depended than Mulliken [64] and smaller in magnitude (relative to Mulliken) [65]. The absolute magnitude of the calculated charges obtained through populations analysis has little physical meaning: These calculations may depend on the method and basis sets used [65], and thus we only focus on the relative changes of these values.

The stabilities of the final geometry conformations are secured via post-geometry optimizations. When Gaussian basis sets are used, *E_ads_* and *E_c_* calculations suffer from basis set superposition errors (BSSE) [66]. The BSSE errors are minimized using large basis sets and employing counterpoise corrections using “ghost” massless atoms in the fragment energy calculations of the metal–graphene structures. Here, we only report BSSE corrected *E_ads_* and *E_c_* values. Calculated *E_ads_* and *E_c_* values using the counterpoise correction are less negative in energy than corresponding uncorrected values [17,49,67].

### 2.3. QTAIM Parameters

The $\rho \left(\vec{r}\right)$ topology is performed by the TOPOND program [68], which is incorporated in CRYSTAL17. TOPOND calculates $\rho \left(\vec{r}\right)$ and its Laplacian ($\nabla^{2}\rho \left(\vec{r}\right))$ at critical points, for periodic and non-periodic systems. The critical points in the $\rho \left(\vec{r}\right)$ and $\nabla^{2}\rho \left(\vec{r}\right)$ topological analyses fulfill the conditions ${\left.\nabla \rho \left(\vec{r}\right)\right|}_{{\vec{r}}_{cp}}$=0 and ${\left.\nabla \left(\nabla^{2}\rho \left(\vec{r}\right)\right)\right|}_{{\vec{r}}_{cp}}$=0, respectively, where ${\vec{r}}_{cp}$ is the vector from the atom to the critical point location. The bond critical points are denoted as (3, -1), where 3 stands for the three non-zero eigenvalues of the $\rho \left(\vec{r}\right)$ Hessian at ${\vec{r}}_{cp}$ and −1 is the algebraic summation of the number of positive and negative eigenvalues. Bonds strengths are better described by (H/$\rho )\left(\vec{r}\right)$ relative to $\rho \left(\vec{r}\right)$ and $\nabla^{2}\rho \left(\vec{r}\right)$, at the bond critical point ${\vec{r}}_{cp}$, where $H\left(\vec{r}\right)=G\left(\vec{r}\right)+V\left(\vec{r}\right)$, $G\left(\vec{r}\right)$ is the positive definite kinetic energy density and $V\left(\vec{r}\right)$ is the potential energy density [69]. Specifically, for covalent bonds, the bond strength and (H/$\rho )\left(\vec{r}\right)$, at the bond critical point, are positively correlated, whereas the opposite is observed for ionic bonds. Moreover, for covalent and ionic bonds, at the bond critical point ${\vec{r}}_{cp}$, (H/$\rho )\left(\vec{r}\right)$ and $\rho \left(\vec{r}\right)$ are positively and negatively correlated, respectively [70,71]. Gatti stated that adoption of a single QTAIM criterion for bond assessment is challenging [70]. Therefore, we used $\rho \left(\vec{r}\right)$, $\nabla^{2}\rho \left(\vec{r}\right)$, and (H/$\rho )\left(\vec{r}\right)$ at metal–C and metal–metal bond critical points ${\vec{r}}_{cp}$, to determine changes in the corresponding bond strengths via QTAIM.

Ionic, covalent, and metallic bonds are classified via changes in $\rho \left(\vec{r}\right)$, $\nabla^{2}\rho \left(\vec{r}\right)$, (H/$\rho )\left(\vec{r}\right)$, and (G/$\rho )\left(\vec{r}\right)$, at bond critical points ${\vec{r}}_{cp}$ [69]. Ionic bonds, such as Li-C and Na-C (vide infra), satisfy the conditions $\nabla^{2}\rho \left({\vec{r}}_{cp}\right)$ > 0, (H/$\rho )\left({\vec{r}}_{cp}\right)>0$, and (G/$\rho )\left({\vec{r}}_{cp}\right)\geq 1$, whereas $\rho \left({\vec{r}}_{cp}\right)$ is small (at Li-C and Na-C bond critical points). Here, the metal–metal bonds appear as non-polar covalent or metallic bonds (vide infra). For non-polar covalent bonds, $\nabla^{2}\rho \left({\vec{r}}_{cp}\right)\ll$ 0, (H/$\rho )\left({\vec{r}}_{cp}\right)\ll 0$, and (G/$\rho )\left(\vec{r}\right)<1$. The C-C bonds have large $\rho \left({\vec{r}}_{cp}\right)$ values, whereas covalent Li-Li bonds, found in small Li clusters, have small $\rho \left({\vec{r}}_{cp}\right)$ values [72]. For metallic bonds, the above conditions are modified as follows: $\rho \left({\vec{r}}_{cp}\right)$ is small, $\nabla^{2}\rho \left({\vec{r}}_{cp}\right)$ ~ 0, and (H/$\rho )\left({\vec{r}}_{cp}\right)<0$. Metallicity $\xi \left(\vec{r}\right)=\frac{\rho \left(\vec{r}\right)}{\nabla^{2}\rho \left(\vec{r}\right)}$ (units of length^2^) serves as an additional criterion to classify non-polar covalent and metallic bonds [73,74]. For metallic bonds, $\xi \left(\vec{r}\right)>1$, whereas negative metallicity values, due to $\nabla^{2}\rho \left(\vec{r}\right)<0$, are indicative of non-polar covalent bonds [73].

### 2.4. ELF Parameters 

The electron localization function (ELF) is an alternative to QTAIM for analyzing chemical bonding [43,75,76,77]. ELF measures the electron localization in atomic and molecular systems and it was originally described by Becke and Edgecombe [41] as follows:

(3) $$\eta \left(\vec{r}\right)=1/\left(1+\chi {\left(\vec{r}\right)}^{2}\right)$$

where $\chi \left(\vec{r}\right)$ is the relevant ELF kernel and $0\leq \eta \left(\vec{r}\right)\leq 1$ [70]. The $\eta \left(\vec{r}\right)=1$ case corresponds to perfect localization, whereas the $\eta \left(\vec{r}\right)=0.5$ to the electron gas like pair probability. The ELF function $\eta \left(\vec{r}\right)$ reveals atomic shell structure and core, similar to $-\nabla^{2}\rho \left(\vec{r}\right)$. However, $-\nabla^{2}\rho \left(\vec{r}\right)$ fails to reveal the shells structure for heavy atoms [78]. Kohout and Savin found that $\eta \left(\vec{r}\right)$ can resolve all shells for all atoms from Li to Sr [79]. Savin et al. [80] interpreted ELF as a local measure of the Pauli repulsion on the kinetic energy $\rho \left(\vec{r}\right)$, and applied ELF to a variety of atoms, molecules, and solids. Specifically, $\eta \left(\vec{r}\right)\;\sim \;1$ is indicative of weaker Pauli repulsion than in the uniform electron gas. ELF gradient analysis reveals attractors (local maxima) and corresponding basins [43]. In QTAIM, basins are regions in space bounded by zero flux surfaces in the gradient of $\rho \left(\vec{r}\right)$ [81]. Basins are classified as core and valance and characterized by their synaptic order (i.e., the number of core basins that share a common boundary [82]). Only core basins contain a nucleus, whereas valence basins are always connected to at least one core basin. At low $\eta \left(\vec{r}\right)$ values only one surface is visualized, whereas at higher $\eta \left(\vec{r}\right)$ basins separate. Therefore, bifurcation (i.e., tree) diagrams [83], with the bifurcation points corresponding to $\eta \left(\vec{r}\right)$ minima, can be used to examine bond types and regions in space, where lone pair electrons are located. The first bifurcation point corresponds the separation of the core and the valance basins from the same atom. The lower the bifurcation point the more localized are the corresponding basins [43]. The ELF 3D isosurfaces are plotted using Chimera [84].

## 3. Results and Discussion

### 3.1. Structural Properties, E_ads_, and E_c_ for Li and Na Adsorbed on Graphene and Graphene Oxide

#### 3.1.1. Adsorption on Pristine Graphene 

At 1/32 monolayer (ML) coverage, Li and Na are adsorbed on the hollow site, in agreement with past reports [5,16,17,18,22]. At the higher coverages 3/32 ML and 5/32 ML, both metals form clusters (Li_3_, Li_5_, Na_3_, and Na_5_, Figure 1b,c,e,f), as expected [30,31,37]. Here, the effect of D2 corrections in our calculated *E_c_* values is small. However, disagreements between the DFT calculations with and without D2 corrections are observed in the *h* values and the metal–metal closest distances (e.g., for Na_5_/graphene, |Δ*h|* = 0.11 Å, Figure 1f). For Li_3_ and Li_5_ adsorptions on pristine graphene, our closest calculated Li-Li distances are 2.85 Å and 2.77 Å, when D2 corrections are included in our calculations (Figure 1b,c). These values are smaller than the bulk Li closest Li-Li distance of 3.04 Å [85]. Similarly, for the Na case, our closest Na-Na distances during adsorption on pristine graphene are less than the bulk Na closest Na-Na distance of 3.72 Å [86]. These are indicative of the presence of stronger Li and Na intra-cluster bonds relative to the ones in their metallic bulks. 

**Figure 4:**
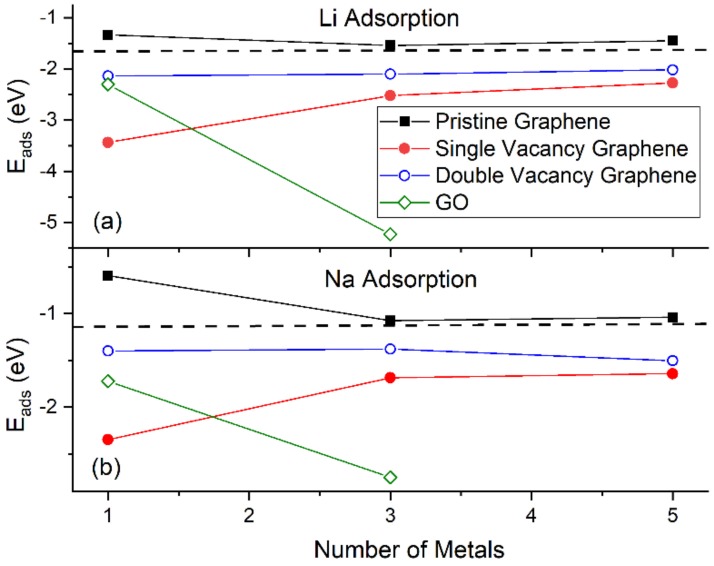
The *E_ads_* for (**a**) Li and (**b**) Na adsorptions on various supports. The horizontal dashed lines are the corresponding bulk metals *E_ads_*. Calculations include D2 correction.

Figure 4 shows the *E_ads_* for Li and Na adsorptions on graphene and GO. At the same metal coverages, Li adsorption on pristine graphene is significantly stronger relative to Na, as shown by *E_ads_*, *E_c_*, and *h* values. This statement is extended for adsorption on defective graphene and GO, with the exception of Na_3_/GO calculations without D2 corrections (vide infra). Our Li and Na |*E_ads_*| values are smaller than the binding energies of bulk Li (1.63 eV/Li [87]) and Na (1.13 eV/Na [88]): These are indicative of unstable Li and Na adsorptions on pristine graphene, at the coverages of this work, in agreement Lee at al. [20] and Liang et al. [37]. Although our calculations are at 0 K, the above metal stability statements are in agreement with experimental observations at room temperature. For example, Na adsorption on pristine graphene has only been detected under Na/C_64_ configuration [89], where in this case Na |*E_ads_*| is larger than its bulk binding energy [37]. For adsorption on pristine graphene, the *E_c_* becomes less negative along with increased metal coverage, thus weakening the metal-support interaction, as expected [17,37]. This statement is extended for adsorption on defective graphene. Table 1 shows our *E_ads_* values and from past reports, for Li and Na adsorbed on pristine and single vacancy graphene: our *E_ads_* values are in good agreement with past work.

#### 3.1.2. Adsorption on Defective Graphene

Li and Na adsorptions on defective graphene are stable (Figure 4). Here, Li and Na |*E_ads_*| values at all coverages are larger than the binding energies of their metallic bulk, in agreement with past reports [20,37]. The presence of defects on graphene significantly strengthens Li and Na adsorption relative to their adsorption on pristine graphene (Figure 1 and Figure 2). Increased carbon vacancies decrease the metal-graphene interaction (Figure 2). For example, for Li/graphene at 1/32 ML coverage, increased C vacancies decrease |*E_c_*| from 3.44 eV to 2.14 eV. At 3/32 ML coverage, the presence of a single C vacancy causes the Li clusters to collapse, whereas in all other cases Li and Na form clusters, in a similar fashion as during adsorption on pristine graphene.

The strong adsorption of Na on defective graphene (and GO, vide infra) is indicative of its possible use in NIB. Although, Na adsorption on graphene is not as strong as Li, its abundancy is a significant advantage over Li.

#### 3.1.3. Adsorption on Graphene Oxide

Li and Na adsorptions on GO are also stable (Figure 4). Moreover, contrary to adsorption on pristine and defective graphene, increased metal coverage strengthens the metal-GO interaction. This statement does not apply for the Na_3_/GO geometry calculated without D2 corrections. Figure 3d,e show striking differences between the optimized geometries for Na_3_/GO with and without D2 corrections. Therefore, for Na adsorption on GO, at coverages > 1/32 ML, it is important to include long-range interactions due to Na atoms’ large polarizations [37]. For Na_3_/GO, Na clustering is observed, in contrast to the Li_3_ case.

### 3.2. Charge transfers and COOP

Table 2 shows the calculated Li and Na Hirshfeld and Mulliken charges and the metal–C COOP values for the smaller metal–C distances per configuration, for Li and Na absorbed on graphene and GO, under the metal coverages examined here. Chan et al. stated that alkalis are very close to ideal ionic bonding and, thus, Li-C and Na-C bonds are expected to be ionic [16]. There is a positive relationship between the average per metal charge transferred to the support and the metal–C bond strength. Both Hirshfeld and Mulliken calculations show that charge is transferred from Li and Na towards the support (graphene or GO). This charge transfer is decreased along with increased metal coverage, in agreement with decreases in the |*E_c_*| and, thus, the weakening of the metal-support interaction.

Dobrota et al. showed that Na adsorption on graphene-OH compounds leads to Na being neutral [96]. This is in contrast with our work, which shows that charge is transferred from Li and Na to GO (Table 2). However, the support of Dobrota et al. represents a reduced GO structure (rGO) with a significantly smaller O/C ratio than in our case.

The metal–C bond strengths can be estimated via changes in their COOP values. The majority of the metal–C COOP values in Table 1 are of the order expected for ionic bonding. However, there are some exceptions: The Li-C COOP value for Li adsorption on single C vacancy graphene (i.e., 0.138) is at the order expected for covalent bonding. Doan et al., using COOP, found that Li bonds covalently to the Nafion SO_3_ group [97]. Our COOP calculations indicate that metal–C bonds, for adsorption on single vacancy graphene, are covalent (Table 1). COOP calculations are basis set dependent [98]. Therefore, these bonds will be also examined via the method and basis set independent QTAIM and ELF analyses in the next sections.

### 3.3. DOS and electronic structures

Figure 5, Figure 6, Figure 7, Figure 8, Figure 9, Figure 10 and Figure 11 show the DOS and band structures for the configurations of Figure 1, Figure 2 and Figure 3, calculated with the inclusion of D2 corrections.

#### 3.3.1. Adsorption on Pristine Graphene

**Figure 5:**
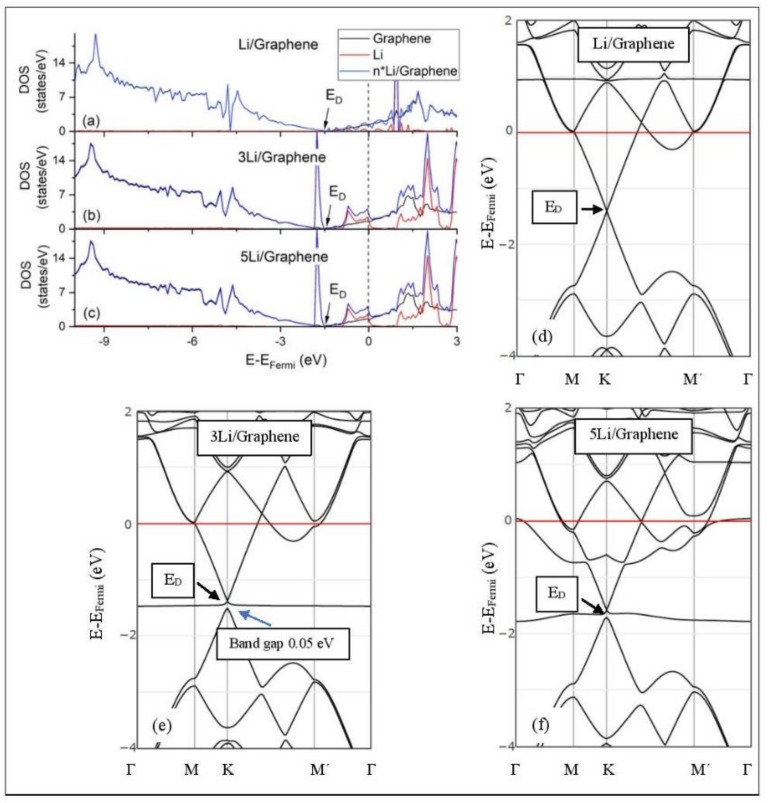
Li adsorption on pristine graphene (**a**–**c**) DOS spectra and (**d**–**f**) band structures. The red horizontal line is the Fermi energy.

**Figure 6:**
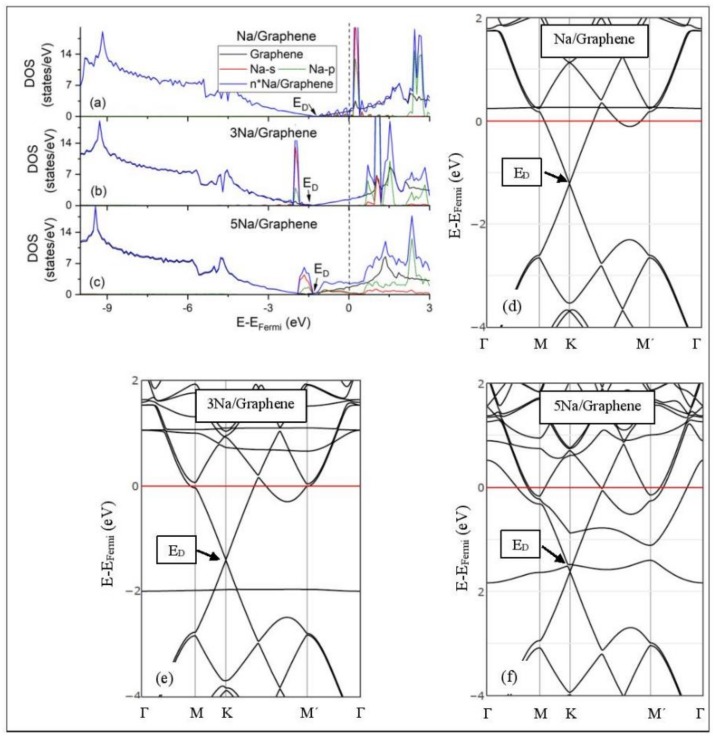
Na adsorption on pristine graphene (**a**–**c**) DOS spectra and (**d**–**f**) band structures. The red horizontal line is the Fermi energy.

The band structure calculations show that Li and Na adsorptions downshift the Dirac point (*E_D_*, DOS(*E_D_*) = 0) below the Fermi energy (Figure 5 and Figure 6). Recall that pristine graphene *E_D_* coincides with the Fermi energy and appears in the band structure as a K-point, where the C_pz_ valance (π) and conduction bands (π*) meet in a linear dispersion fashion [8]. Therefore, pristine graphene is a zero-gap semiconductor (i.e., semi-metal) [99]. The *E_D_* downshift, due to Li and Na adsorptions on pristine graphene, is indicative of n-doped graphene support via charge transfer from Li and Na. At low Li and Na coverages, the *E_D_* is intact (Figure 5d and Figure 6d) and the Li-2s and Na-3s valance orbitals appear in the band structures as flat bands near and above the Fermi energies. Moreover, the DOS spectra below the Fermi energy are unaffected of the adsorption (Figure 5a and Figure 6a), thus supporting a Columbic interaction between the metals and pristine graphene. The DOS and band structure calculations show that Li/graphene and Na/graphene are metallic.

At higher Li coverages, the *E_D_* is broken (Figure 5e,f), whereas for the Na case the *E_D_* is only broken at the 5/32 ML Na coverage (Figure 6f). However, only for the Li_3_/graphene case, a small band gap of 0.05 eV is observed in the *E_D_* region (Figure 5e,f and Figure 6f), turning Li_3_/graphene to a semiconductor. However, this effect may be an artifact of the calculations and it will be further examined in the future. All other metal-pristine graphene configurations are metallic. The flat bands below the Fermi energy in Figure 5e and Figure 6e and the wavy bands in Figure 5f and Figure 6f represent Li-2s, Na-3s, and Na-2p valence states. In the DOS spectrum, these bands appear as peaks near and below the Fermi energy (for Na_5_ case this peak is broadened) and thus, partially occupied. The partial occupancy of these states decreases the Coulombic interaction between the metals and pristine graphene, as the metal coverage increases. This statement also applies for adsorption on defective graphene.

#### 3.3.2. Adsorption on Defective Graphene.

**Figure 7:**
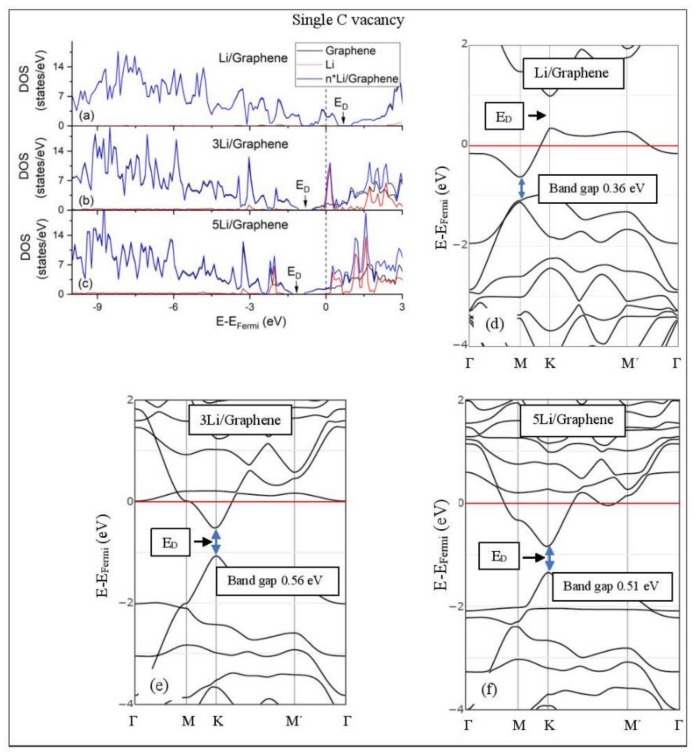
Li adsorption on defective graphene with a single vacancy per 32 C (**a**–**c**) DOS spectra and (**d**–**f**) band structures. The red horizontal line is the Fermi energy. Blue arrows show band gaps.

**Figure 8:**
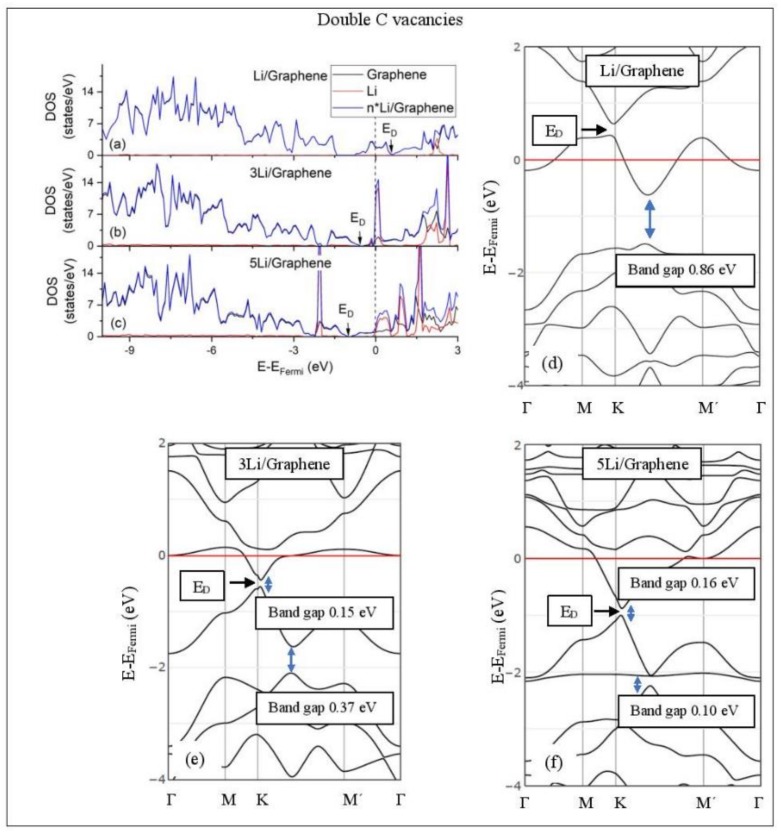
Li adsorption on defective graphene with double vacancies per 32 C (**a**–**c**) DOS spectra and (**d**–**f**) band structures. The red horizontal line is the Fermi energy. Blue arrows show band gaps.

**Figure 10:**
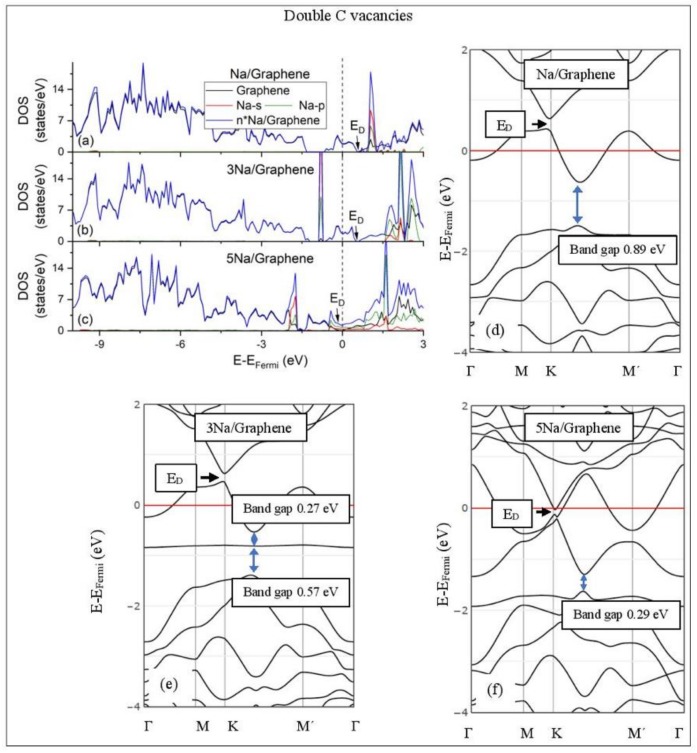
Na adsorption on defective graphene with double vacancies per 32 C (**a**–**c**) DOS spectra and (**d**–**f**) band structures. The red horizontal line is the Fermi energy. Blue arrows show band gaps.

Defects on graphene upshift the *E_D_* above the Fermi energy and create band gaps. For example, the double vacancy graphene is a semiconductor [37]. This *E_D_* upshift is indicative of a p-doped graphene support [37]. At low Li and Na coverages and for Na_3_ adsorbed on double vacancy graphene, the *E_D_* appears above the Fermi energy (Figure 7d, Figure 8d, and Figure 10e). Liang et al. showed that doping defective graphene with Na ions downshifted *E_D_*, but still it remained above the Fermi energy. Therefore, Li and Na adsorptions on defective graphene decrease p-doping. Moreover, at low metal coverages, the observed band gaps further grow as vacancies increase. The DOS spectra (Figure 7a and Figure 8a) show minimal hybridization of the metal valance states with the graphene bands, which supports metal-graphene Coulombic interaction. However, we will show in the next section that the Li-C bond for Li adsorbed on single vacancy graphene has some covalent character.

With the exception of Na_3_ adsorbed on double vacancy graphene, increased Li and Na coverages on defective graphene push the *E_D_* below the Fermi energy, thus changing graphene to n-doped. For Li adsorption on single vacancy graphene the band gap grows due to Li clustering, whereas the opposite is observed for the Na case. Multiple band gaps are observed for select Li and Na clusters adsorbed on double vacancy graphene. Here, all metal-defective graphene configurations are semiconductors. Our Na band structure calculations are in agreement with Liang et al. [37].

#### 3.3.3. Adsorption on GO

**Figure 11:**
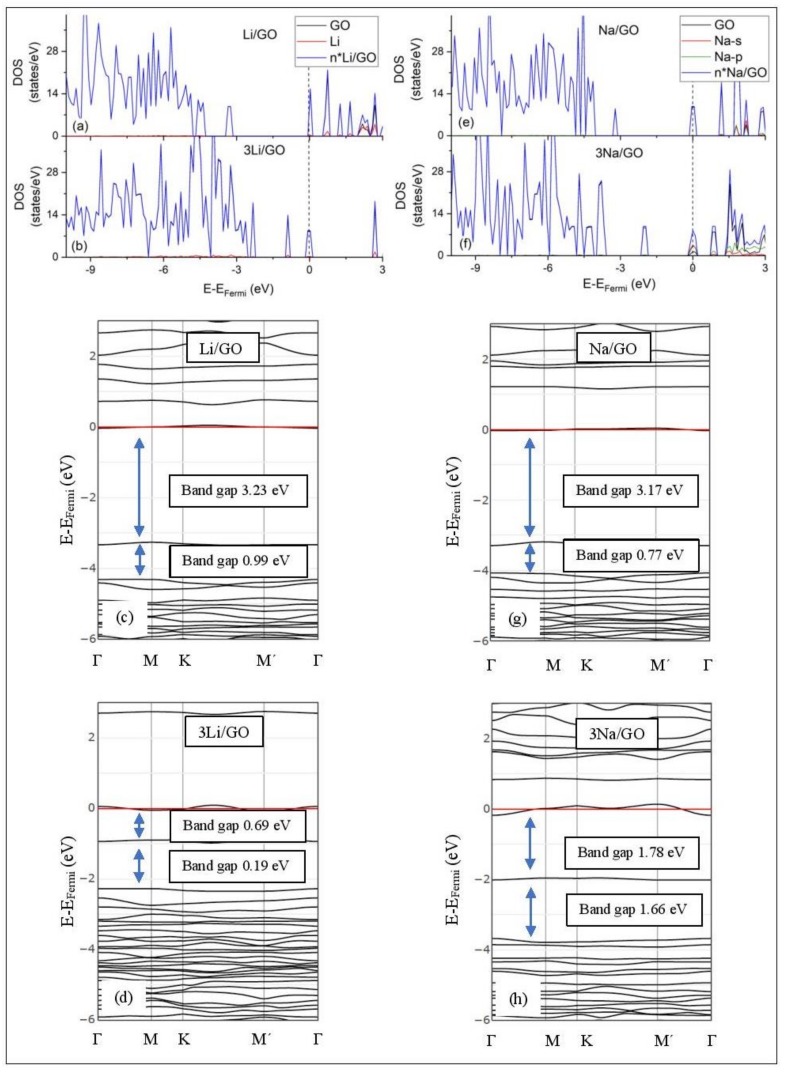
DOS spectra and band structures for adsorption on GO (**a**–**d**) Li and (**e**–**h**) Na. The red horizontal line is the Fermi energy. Blue arrows show band gaps close to the Fermi energy.

Graphene Oxide, at O/C = 43.75%, is an insulator. Its calculated band gap is 5.26 eV, when only epoxides are present in the GO structure (PBE0 calculations without D2 correction) [50]. This value is decreased to 4.40 eV, for GO that includes both epoxides and hydroxyls, under the same DFT calculations. The GO band structure calculations do not reveal *E_D_* (Figure 11). Li and Na adsorption on GO is associated with multiple band gaps below the Fermi energy. For example, the largest of these band gaps are 3.23 eV and 3.17 eV for Li/GO and Na/GO, respectively. The largest band gap width and the Li and Na coverages are negatively correlated (Figure 11). The GO DOS spectra below the Fermi energy show minimal contributions from the metals valance states. However, the Li/GO and Na/GO DOS spectra below the Fermi energy are different, which implies structural changes on GO support due to metal adsorption. This statement also applies to adsorptions at higher metal coverages.

### 3.4. QTAIM analysis

**Figure 12:**
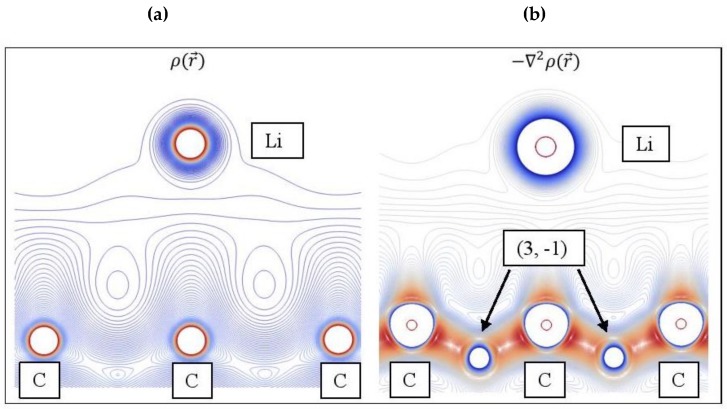
(**a**) Contour plots for Li/graphene electron density $\rho \left(\vec{r}\right)$. (**b**) The negative of its Laplacian $-\nabla^{2}\rho \left(\vec{r}\right)$, which reveals saddle points (3, -1). Colors as follows: Red: High $\rho \left(\vec{r}\right)$; Blue: Low $\rho \left(\vec{r}\right)$.

Figure 12 shows the $\rho \left(\vec{r}\right)$ and $-\nabla^{2}\rho \left(\vec{r}\right)$ contour plots for Li adsorbed on pristine graphene. The topology of the Laplacian is given by $-\nabla^{2}\rho \left(\vec{r}\right)$, since charge is concentrated at $\nabla^{2}\rho \left(\vec{r}\right)$ < 0. The $-\nabla^{2}\rho \left(\vec{r}\right)$ reveal altering shells of charge concentrations and depletions (valence shell charge concentration-VSCC [100]) and (3, -1) saddle points, whereas this information is absent in $\rho \left(\vec{r}\right)$ (Figure 12a). Aray and Rodriguez [101] stated that maxima are linked with other maxima by trajectories of $-\nabla^{2}\rho \left(\vec{r}\right)$ originating at (3, -1) saddle points. Evidently, the (3, -1) saddle points that connect Li and C are not shown in Figure 12.

#### 3.4.1. The Metal–C Bond

Table 3 and Table 4 show the QTAIM properties $\rho \left(\vec{r}\right)$, $\nabla^{2}\rho \left(\vec{r}\right)$, and (G/$\rho )\left(\vec{r}\right)$ at the Li-C and C-Na bond critical points, respectively (closest metal–C distances). Moreover, for adsorption on GO, the above QTAIM properties are also evaluated at the metal-O bond critical points. Table 3 shows that some Li-C bond critical points for Li_3_ adsorption on pristine graphene and GO are not revealed by TOPOND. Moreover, for the former case, the Li-C critical point at 2.28 Å appears as a (3, +1) point (i.e., charge minima). In all cases here, with the exception of Li adsorbed on single vacancy graphene, QTAIM shows that metal–C bonds are ionic. Recall that for ionic bonding $\nabla^{2}\rho \left({\vec{r}}_{cp}\right)$ > 0, (H/$\rho )\left({\vec{r}}_{cp}\right)>0$, and (G/$\rho )\left({\vec{r}}_{cp}\right)\geq 1$, whereas $\rho \left({\vec{r}}_{cp}\right)$ is small. The Li-C bond, for Li adsorption on single vacancy graphene, is analyzed using the bond classification of Espinosa et al. [71]. Here (|V|/$G)\left({\vec{r}}_{cp}\right)<1$ is indicative of ionic bonding and (|V|/$G)\left({\vec{r}}_{cp}\right)=1$ is the lower limit of the transit region indicating incipient covalent bond formation. Recall that for this case, the Li-C COOP value is 0.138, and is the highest value observed in this work for metal–C bonds. Therefore, the presence of vacancies in the support may add a covalent character in the metal–C bonds.

**Figure 13:**
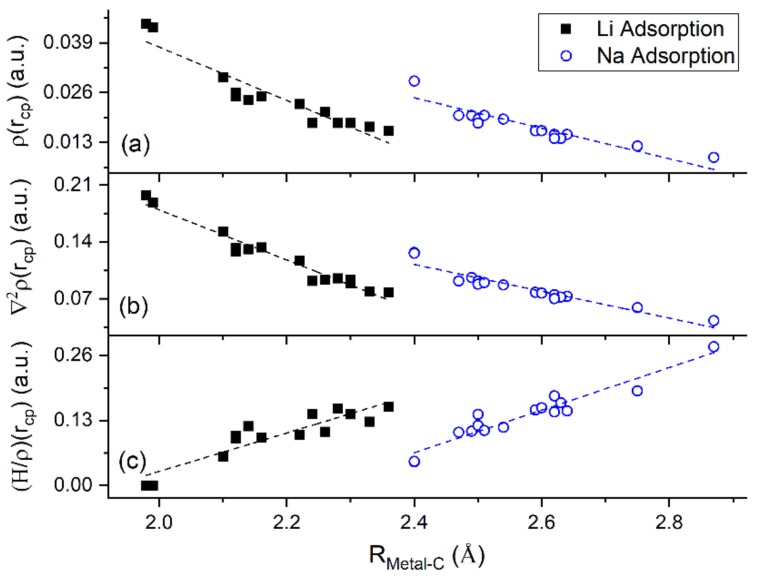
QTAIM parameters vs. metal–C distance for Li and Na adsorbed on graphene and GO. (**a**) $\rho \left(\vec{r}\right)$, (**b**) $\nabla^{2}\rho \left(\vec{r}\right)$, and (**c**) (H/$\rho )\left(\vec{r}\right)$ at metal–C bond critical points (cp).

QTAIM shows that for adsorption on GO, Li and Na form ionic bonds of similar strength with both C and O atoms. This explains the significantly stronger metal-GO interaction relative to adsorption on pristine graphene, at the same metal coverages. Figure 13 shows a negative correlation between (H/$\rho )\left(\vec{r}\right)$ and $\rho \left(\vec{r}\right)$ thus, verifying ionic metal–C bonding.

#### 3.4.2. The Metal–metal Bond

Table 5 and Table 6 show the QTAIM properties $\rho \left(\vec{r}\right)$*, (H/*$\rho )\left(\vec{r}\right)$, *(G/*$\rho )\left(\vec{r}\right)$, $\xi \left(\vec{r}\right)$, and the sign of $\nabla^{2}\rho \left(\vec{r}\right)$ and ELF $\eta \left(\vec{r}\right)$ values at the Li-Li and Na-Na bond critical points, respectively. Not all metal–metal bond critical points are revealed for both Li and Na clusters adsorbed on graphene and GO (Table 4 and Table 5). Moreover, the Li-Li bond critical points are not revealed for Li_3_ adsorbed on defective graphene and for Li_3_ and Li_5_ adsorbed on GO. Recall that for metallic bonds ξ>1, whereas ξ<0, due to ρ<0, is indicative of non-metallic non-polar covalent bonds. For Li_3_ adsorbed on pristine graphene, Na_3_ adsorbed on pristine and double vacancy graphene, and for Li_5_ and Na_5_ adsorbed on defective graphene all intra metal-metal bonds are metallic (ξ>1, Table 3). However, Li_5_, Na_3_, Na_5_ show a mixture of covalent and metallic metal-metal bonds, when are adsorbed on other supports (e.g., Li_5_ on pristine graphene, Table 5). Therefore, the support selection during metal cluster adsorption could alter the metal-metal bond types.

### 3.5. ELF Analysis

Ayers and Jenkins stated that metallic bonds rarely have ELF $\eta \left(\vec{r}\right)$ values larger than 0.6 [73]. Our $\eta \left(\vec{r}\right)$ values, for metallic metal–metal bonds at their bond critical points, are less than 0.43, which is within the Ayers and Jenkins ELF limit for metallic bonds. However, some Li-Li and Na-Na non-metallic covalent bonds have low $\eta \left(\vec{r}\right)$ values comparable to the ones for metallic bonds. For example, for Li_5_ adsorbed on pristine graphene the Li-Li bond with bond length 2.91 Å has a negative metallicity but its $\eta \left(\vec{r}\right)$ value is 0.39. A similar case appears for Na_3_/GO.

**Figure 14:**
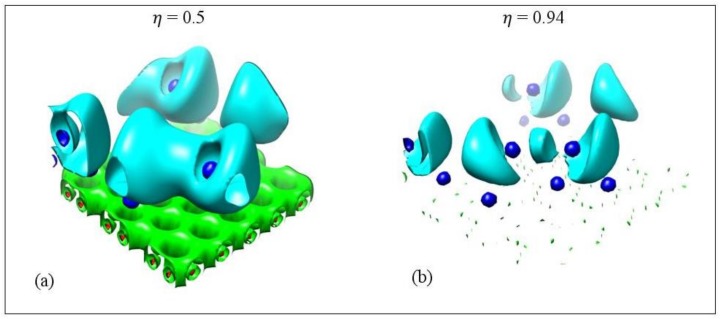
Electron localization isosurfaces for Li_5_ adsorbed on pristine graphene at ELF values. (**a**) 0.5 and (**b**) 0.94. Colors are as follows: C(Li) blue, C(C) red, V(Ci,Cj) green, and V(Li) and V(Li,Li) cyan. Calculations include D2 correction.

**Figure 15:**
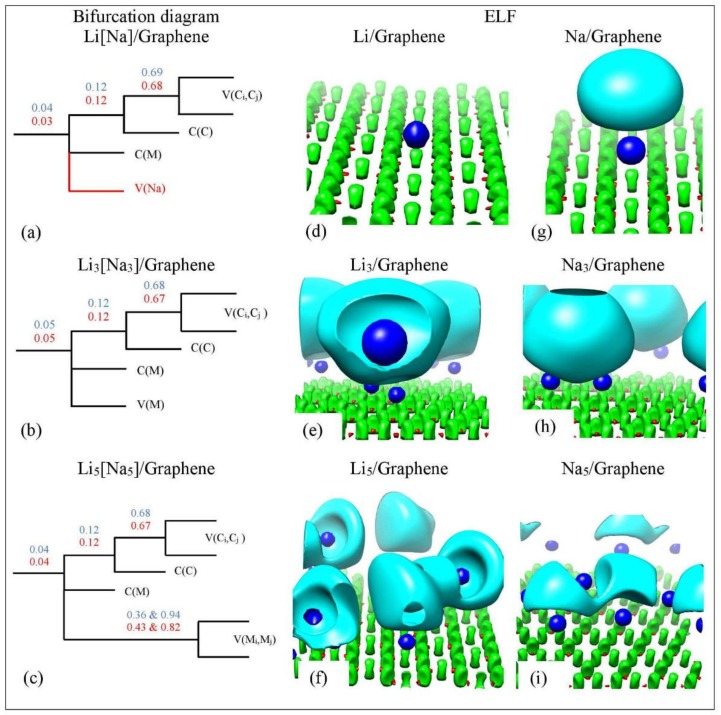
(**a**–**c**) Bifurcation diagrams for Li_n_ and Na_n_ *n* = 1, 3, 5 adsorbed on pristine graphene. Colors are as follows: blue and red refer to Li and Na adsorption, respectively and black refers to both metals. (**d**–**i**) corresponding ELF isosurfaces at ELF = 0.75. Colors are as follows: C(M) blue, C(C) red, V(Ci,Cj) green, and V(M) and V(M,M) cyan, M = Li, Na. Calculations include D2 correction.

**Figure 16:**
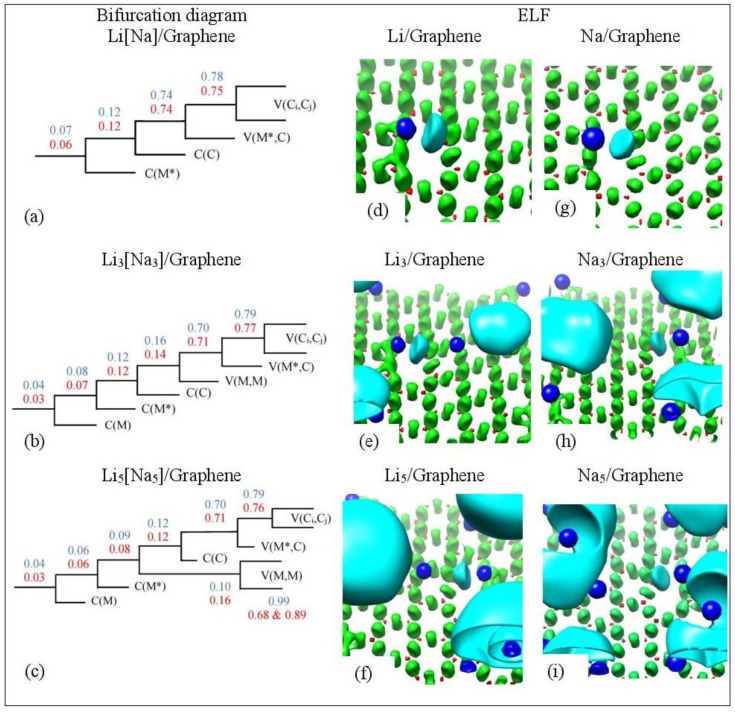
(**a**–**c**) Bifurcation diagrams for Lin and Nan *n* = 1, 3, 5 adsorbed on single vacancy defective graphene. Colors are as follows: blue and red refer to Li and Na adsorption, respectively, and black refers to both metals. (**d**–**i**) Corresponding ELF isosurfaces at ELF = 0.75. Colors are as follows: C(M) blue, C(C) red, V(Ci,Cj) green, and V(M) and V(M,M) cyan, M = Li, Na. M* is the metal atom close to the defect. Calculations include D2 correction.

Figure 14 shows the ELF isosurfaces for Li_5_ adsorbed on pristine graphene at $\eta \left(\vec{r}\right)$ values 0.5 and 0.94. Recall that ELF analyzes chemical bonding by yielding basins of attractors. At $\eta \left(\vec{r}\right)$ = 0.5 the Li-Li valance basin (cyan surface) surrounds several Li atoms, whereas at $\eta \left(\vec{r}\right)$ = 0.94 it breaks in smaller isosurfaces. Figure 15 and Figure 16 show the bifurcation diagrams and corresponding isosurfaces for Li and Na adsorbed on pristine and single vacancy graphene, respectively. The values shown in bifurcation diagrams correspond to the $\eta \left(\vec{r}\right)$ value, where a separation between the basins occurs. We start our ELF analyzes with Li and Na adsorption on pristine graphene, at low metal coverages.

For Li/graphene, the bifurcation diagram reveals the Li and C core basins (C(Li) and C(C)) and the polysynaptic valence basin V(C,C) (Figure 15a). For Na/graphene, the bifurcation diagram additionally reveals a monosynaptic valance V(Na) (Figure 15g). For Li/graphene, the first bifurcation occurs at $\eta \left(\vec{r}\right)$ = 0.04, where C(Li) separates from the disynaptic basin. Here, the second bifurcation occurs at $\eta \left(\vec{r}\right)$ = 0.12, for the C(C) separation, whereas the V(C,C) separation appears at $\eta \left(\vec{r}\right)=0.69$. Similar $\eta \left(\vec{r}\right)$ separation values are found for Na/graphene. Our ELF separation values are in agreement with those found by Matito et al. [81], who studied the bonding in methylalkalimetals (CH_3_M)_n_ (M = Li, Na, and K; *n* = 1, 4). The $\eta \left(\vec{r}\right)$ C(Li) and C(Na) separation values are indicative of ionic bonding between Li and Na metals and C, in agreement with QTAIM and electronic structure analysis (vide supra). It is noteworthy that our C(Na) $\eta \left(\vec{r}\right)$ separation values are never higher than C(Li) values, in agreement with the weaker Na-graphene interaction relative to Li-graphene (Figure 15 and Figure 16). At higher metal coverages, ELF reveals monosynaptic V(Li) and V(Na) (Figure 15b) and polysynaptic V(Li,Li) and V(Na,Na) basins (Figure 15c).

At low metal coverages and for adsorption on single vacancy graphene, the first bifurcation point occurs at higher $\eta \left(\vec{r}\right)$ values relative to adsorption on pristine graphene. These higher $\eta \left(\vec{r}\right)$ separation values are indicative of stronger metal–C bonding and are in the covalent bond region (Figure 16a). COOP, QTAIM, and ELF agree that the Li-C bond, for adsorption on single vacancy graphene, has some covalent character. However, the Na-C covalent character, for Na adsorbed on single vacancy graphene, indicated by ELF analyses is not agreement with QTAIM, whereas it agrees with COOP. Disparagements between ELF and QTAIM have been reported [81].

## 4. Conclusions

Li and Na adsorptions on pristine and defective graphene and GO has been studied using periodic DFT structural and electronic calculations paired with COOP, QTAIM, and ELF analyzes. DFT optimized geometries show that increased metal coverage clusters Li and Na during adsorption on graphene, which lowers the efficiency of LIB and NIB. However, Li_3_ clusters collapse during adsorption on single vacancy graphene and GO. The *E_ads_* calculations show that Li and Na adsorption on pristine graphene is unstable at all the metal coverages examined here, whereas the presence of defects in the graphene support stabilizes Li and Na adsorptions. Increased metal coverage weakens metal-graphene and strengthen metal-GO interactions. Adsorption on GO is strong due to simultaneous metal bonding with C and O GO atoms.

DOS calculations show that, at low metal coverages, Li and Na adsorptions on pristine does not affect the DOS spectra below the Fermi energy. This effect is indicative of Coulombic interactions between the adsorbates and the support. These interactions weaken as metal coverages increase, due to the downshift of the metal valances below the Fermi energy. The band calculations show that the *E_D_* is pushed below the Fermi energy due to Li and Na adsorptions on pristine graphene and may be associated with small band gaps at higher metal coverages. The presence of defects on graphene is associated with *E_D_* shifting and bang gap openings.

The metal–C bonds are analyzed using COOP, QTAIM, and ELF. The COOP metal–C COOP values at the order expected for ionic bonding, for all cases except for adsorption on single vacancy graphene, at low metal coverages. QTAIM shows that only the Li-C bond contains covalent character for adsorption on single vacancy graphene, whereas in all other cases the metal–C bond is ionic. ELF partially agrees with QTAIM and shows that both Li-C and Na-C bonds are covalent for adsorption on single vacancy graphene. QTAIM and EFT are also used to study Li-Li and Na-Na bonds: Li and Na clusters may contain metallic and covalent metal–metal bonds depending on adsorption support (i.e., pristine and graphene and GO).

## Figures and Tables

**Figure 9:**
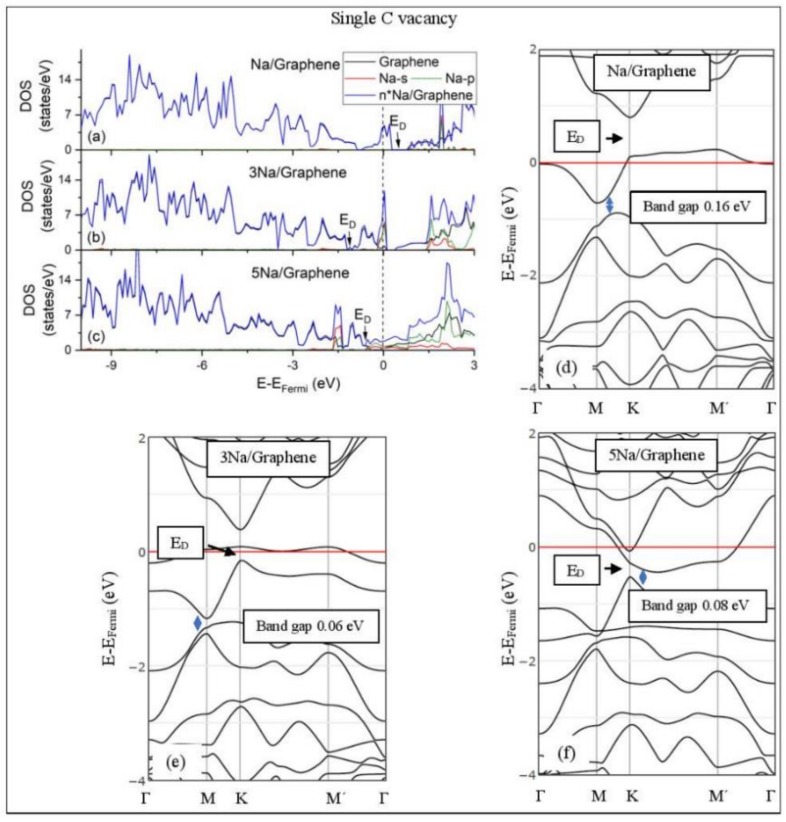
Na adsorption on defective graphene with single vacancy per 32 C. (**a**–**c**) DOS spectra and (**d**–**f**) band structures. The red horizontal line is the Fermi energy. Blue arrows show band gaps.

**Table 1:** Calculated *E_ads_* for Li and Na adsorbed on graphene compared with past work. Values in parentheses include D2 correction. Unless otherwise stated, the graphene support contains 32 atoms per unit cell (4 × 4 supercell).

System	Vacancies	Current Work	Past work	
			*E_ads_* (eV)	Method
Li /Graphene	0	−1.34 (−1.33)	−1.19 [90], −1.096 [16]	PBE [91]
			−1.36 [92]	LDA
			−1.56 [90]	PBE+D2 [54,91]
			−1.23 [90]	PBE+D3 [91,93]
			−1.05 [90]	PBE+vdW-DF2 [91]
	1	(−3.44)	−2.65 [94]	PBE [91]
			−2.94 [94]	PBE+D2 [54,91]
			−2.72 [94]	PBE+D3 [91,93]
			−2.59 [94]	PBE+vdW-DF2 [91,95]
Na /Graphene	0	−0.59 (−0.59)	−0.55 [90], −0.462 [16]	PBE [91]
			−0.72 [92] ^a^	LDA
			−0.93 [90]	PBE+D2 [54,91]
			−0.64 [90]	PBE+D3 [91,93]
			−0.49 [90]	PBE+vdW-DF2 [91,95]
			−0.88 [37]	PBE+D2 [91]
	1	(−2.35)	−1.88 [94]	PBE [91]
			−2.20 [94]	PBE+D2 [54,91]
			−2.00 [94]	PBE+D3 [91,93]
			−1.74 [94]	PBE+vdW-DF2 [91,95]

^a^ 3 × 3 supercell.

**Table 2:** Li and Na Hirshfeld and Mulliken electronic charges (average values per metal) and metal–C COOP for the smaller metal–C distances, for Li and Na absorbed on graphene and GO. Values in parentheses include D2 correction.

Metals	Support	Vacancies	Metal charge (e)		Metal–C COOP ^a^
			Hirshfeld	Mulliken	
Li	Graphene	0	2.27 (2.27)	2.66 (2.64)	0.041 (0.040)
		1	(2.37)	(2.73)	(0.138) ^3^
		2	(2.31)	(2.67)	(0.039) ^6^
	GO	0	2.13 (2.14)	2.43 (2.45)	0.085 (0.082) ^1^
Li_3_	Graphene	0	2.98 (2.98)	2.97 (2.97)	0.024 (0.024)
		1	(2.64)	(2.84)	(0.036) ^6^
		2	(2.76)	(2.84)	(0.006) ^4^
	GO	0	2.12 (2.13)	2.42 (2.43)	0.088 (0.090) ^1^
Li_5_	Graphene	0	3.01 (3.02)	2.99 (2.99)	0.015 (0.022)
		1	(2.81)	(2.91)	(0.029) ^6^
		2	(2.84)	(2.43)	(0.036) ^6^
Na	Graphene	0	10.53 (10.53)	10.81 (10.58)	0.043 (0.042)
		1	(10.32)	(10.76)	(0.116) ^3^
		2	(10.21)	(10.68)	(0.043) ^6^
	GO	0	10.13 (10.13)	10.53 (10.54)	0.051(0.050) ^1^
Na_3_	Graphene	0	10.85 (10.85)	10.97 (10.98)	0.039 (0.039)
		1	(10.72)	(10.97)	(0.039) ^6^
		2	(10.85)	(10.97)	(0.035) ^6^
	GO	0	10.72 (10.52)	10.84 (10.64)	0.018 (0.032)
Na_5_	Graphene	0	10.95 (10.95)	11.03 (11.04)	0.031 (0.037) ^6^
		1	(10.85)	(11.01)	(0.039) ^6^
		2	(10.86)	(11.00)	(0.035) ^6^

^a^ Superscript: Number of metal–C COOP values included in the calculations, i = 1−6.

**Table 3:** QTAIM parameters $\rho \left(\vec{r}\right)$, $\nabla^{2}\rho \left(\vec{r}\right)$, (H/$\rho )\left(\vec{r}\right)$, and (G/$\rho )\left(\vec{r}\right)$ at the Li-C bond critical points for the shortest Li-C distances, for Li adsorbed on graphene, with and without vacancies, and GO. For adsorption on GO, these properties are also calculated at the Li-O bond critical point. Values in parentheses include D2 correction.

Metals	Support	Vacancies	Distances (Å)	QTAIM properties (a.u.)			
			Li-C [Li-O]	$\rho \left(\vec{r}\right)$	$\nabla^{2}\rho \left(\vec{r}\right)$	$(H/\rho )\left(\vec{r}\right)$	$(G/\rho )\left(\vec{r}\right)$
Li	Graphene	0	2.16	0.025	0.133	0.095	1.224
			(2.22)	(0.023)	(0.117)	(0.101)	(1.192)
		1	(1.98)	(0.044)	(0.197)	(< 0.01)	(1.111)
		2	(2.12)	(0.026)	(0.132)	(0.094)	(1.185)
	GO	0	2.33	0.017	0.079	0.127	1.020
			(2.26)	(0.021)	(0.093)	(0.107)	(1.000)
			[(1.84)]	[(0.039)]	[(0.276)]	[(0.136)]	[(1.569)]
Li_3_	Graphene	0	2.28^1^	0.022^1^	0.136^1^	0.188^1^	1.325^1^
			(2.30)	(0.018)	(0.093)	(0.142)	(1.147)
		1	(2.14)	(0.024)	(0.131)	(0.118)	(1.228)
		2	(2.12)	(0.025)	(0.128)	(0.099)	(1.162)
	GO	0	2.36^2^	0.016	0.078	0.157	1.057
			(2.30)	(0.018)	(0.089)	(0.143)	(1.068)
			[(1.82)]	[(0.042)]	[(0.294)]	[(0.186)]	[(1.565)]
Li_5_	Graphene	0	2.24	0.018	0.092	0.143	1.131
			(2.28)	(0.018)	(0.095)	(0.153)	(1.155)
		1	(1.99)	(0.043)	(0.188)	(< 0.01)	(1.098)
		2	(2.10)	(0.030)	(0.153)	(0.057)	(1.209)

^1^ Values are for (3, +1) cp. ^2^ Bond critical point for d_Li-C_ = 2.31 Å was not revealed.

**Table 4:** QTAIM parameters $\rho \left(\vec{r}\right)$, $\nabla^{2}\rho \left(\vec{r}\right)$, (H/$\rho )\left(\vec{r}\right)$, and (G/$\rho )\left(\vec{r}\right)$ at the Na-C bond critical points for the shortest Na-C distances, for Na adsorbed on graphene, with and without vacancies, and GO. For adsorption on GO, these properties are also calculated at the Li-O bond critical point. Values in parentheses include D2 correction.

Metals	Support	Vacancies	Distances (Å)	QTAIM properties (a.u.)			
			Na-C[Na-O]	$\rho \left(\vec{r}\right)$	$\nabla^{2}\rho \left(\vec{r}\right)$	$(H/\rho )\left(\vec{r}\right)$	$(G/\rho )\left(\vec{r}\right)$
Na	Graphene	0	2.62	0.015	0.075	0.147	1.105
			(2.64)	(0.015)	(0.073)	(0.149)	(1.098)
		1	(2.47)	(0.020)	(0.092)	(0.106)	(1.054)
		2	(2.49)	(0.020)	(0.096)	(0.108)	(1.108)
	GO	0	2.54	0.019	0.087	0.116	1.048
			(2.50)	(0.019)	(0.092)	(0.120)	(1.074)
			[(2.37)]	[(0.019)]	[(0.162)]	[(0.237)]	[(1.321)]
Na_3_	Graphene	0	2.50	0.018	0.088	0.142	1.093
			(2.59)	(0.016)	(0.078)	(0.151)	(1.085)
		1	(2.40)	(0.029)	(0.127)	(0.047)	(1.031)
		2	(2.63)	(0.014)	(0.072)	(0.165)	(1.083)
	GO	0	2.87	0.009	0.043	0.277	1.005
			(2.60)	(0.016)	(0.077)	(0.155)	(1.048)
			[(2.27)]	[(0.023)]	[(0.152)]	[(0.237)]	[(1.426)]
Na_5_	Graphene	0	2.75	0.012	0.059	0.189	1.027
			(2.62)	(0.014)	(0.070)	(0.179)	(1.067)
		1	(2.40)	(0.029)	(0.126)	(0.048)	(1.035)
		2	(2.51)	(0.020)	(0.090)	(0.110)	(1.001)

**Table 5:** QTAIM parameters $\rho \left(\vec{r}\right)$, (H/$\rho )\left(\vec{r}\right)$, (G/$\rho )\left(\vec{r}\right)$, and $\xi \left(\vec{r}\right)$ and ELF at all available Li-Li bond critical points, for Li adsorbed on graphene, with and without vacancies, and GO. Here, $\left|\nabla^{2}\rho \left(\vec{r}\right)\right|<0.01.$ Values in parentheses include D2 correction.

System	Vacancies	Li-Li (Å)	QTAIM properties (a.u.)				ELF
			$\rho \left(\vec{r}\right)$	$(H/\rho )\left(\vec{r}\right)$	$(G/\rho )\left(\vec{r}\right)$	$\xi \left(\vec{r}\right)$	$\eta \left(\vec{r}\right)$
Li_3_/Graphene	0	2.92	0.013^1^	−0.159	0.233	3.37	0.31
		2.92	0.013^1^	−0.158	0.234	3.28	0.31
		(2.85)	(0.014)^1^	(−0.166)	(0.243)	(3.25)	(0.31)
		(2.85)	(0.014)^1^	(−0.165)	(0.243)	(3.21)	(0.31)
Li_5_/Graphene	0	2.73	0.011^2^	−0.174	0.093	−3.09	0.69
		2.97	0.011^1^	−0.150	0.228	3.20	0.28
		3.04	0.013^1^	−0.153	0.249	2.55	0.30
		(2.77)	(<0.01)^2^	(−0.148)	(0.044)	(−2.40)	(0.89)
		(2.91)	(0.010)^2^	(−0.170)	(0.164)	(−40.60)	(0.39)
		(2.92)	(0.012)^1^	(−0.163)	(0.211)	(5.27)	(0.34)
	1	(2.70)	(<0.01)^1^	(−0.115)	(0.227)	(2.24)	(0.22)
		(2.84)	(0.011)^1^	(−0.159)	(0.215)	(4.24)	(0.32)
		(3.18)	(<0.01)^1^	(−0.128)	(0.246)	(2.13)	(0.21)
	2	(2.87)	(0.010)^1^	(−0.152)	(0.214)	(4.07)	(0.29)
		(3.37)	(<0.01)^1^	(−0.115)	(0.251)	(1.84)	(0.18)
		(3.49)	(<0.01)^1^	(−0.087)	(0.233)	(1.71)	(0.11)

^1^$\nabla^{2}\rho \left(\vec{r}\right)$ > 0 and ^2^ $\nabla^{2}\rho \left(\vec{r}\right)$ < 0.

**Table 6:** QTAIM parameters $\rho \left(\vec{r}\right)$, (H/$\rho )\left(\vec{r}\right)$, (G/$\rho )\left(\vec{r}\right)$, and $\xi \left(\vec{r}\right)$ and ELF at all available Na-Na bond critical points, for Na adsorbed on graphene, with and without vacancies, and GO. Here, $\left|\nabla^{2}\rho \left(\vec{r}\right)\right|<0.01.$ Values in parentheses include D2 correction.

System	Vacancies	Na-Na (Å)	QTAIM properties (a.u)				ELF
			$\rho \left(\vec{r}\right)$ (×10)	$(H/\rho )\left(\vec{r}\right)$	$(G/\rho )\left(\vec{r}\right)$	$\xi \left(\vec{r}\right)$	$\eta \left(\vec{r}\right)$
Na_3_/Graphene	0	3.35	0.09^1^	−0.103	0.186	3.02	0.31
		3.37	0.09^1^	−0.096	0.197	2.50	0.28
		(3.23)	(0.10)^1^	(−0.109)	(0.205)	(2.60)	(0.30)
		(3.24)	(0.10)^1^	(−0.108)	(0.207)	(2.51)	(0.29)
	1	(3.82)^3^	(0.04)^2^	(−0.124)	(0.054)	(−3.59)	(0.65)
	2	(3.35)	(0.08)^1^	(−0.099)	(0.190)	(2.75)	(0.28)
		(3.38)	(0.08)^1^	(−0.093)	(0.198)	(2.38)	(0.26)
Na_3/_GO	0	3.34	0.09^1^	−0.097	0.184	2.84	0.30
		3.43	0.08^1^	−0.088	0.195	2.33	0.26
		(3.19)	0.05^2^	(−0.151)	(0.137)	(−17.51)	(0.28)
Na_5/_Graphene	0	3.66	0.06^2^	−0.114	0.061	-4.71	0.71
		4.10	0.06^1^	−0.104	0.114	26.06	0.43
		(3.54)	0.07^2^	(−0.121)	(0.029)	(−2.71)	(0.93)
		(4.05)	(0.07)^1^	(−0.110)	(0.141)	(8.04)	(0.34)
	1	(3.55)	(0.06)^1^	(−0.113)	(0.115)	(201.62)	(0.41)
		(3.63)	(0.06)^1^	(−0.089)	(0.151)	(4.017)	(0.28)
	2	(3.34)	(0.07)^1^	(−0.084)	(0.220)	(1.84)	(0.20)
		(3.41)	(0.07)^1^	(−0.090)	(0.182)	(2.70)	(0.26)

^1^$\nabla^{2}\rho \left(\vec{r}\right)$ > 0 and ^2^ $\nabla^{2}\rho \left(\vec{r}\right)$ < 0. ^3^ Bond cp for d_Na-Na _ = 3.77 Å was not revealed.
